# Letter to the Editor: “Circulating Cell-Free DNA and Circulating Tumor Cells as Prognostic and Predictive Biomarkers in Advanced Non-Small Cell Lung Cancer Patients Treated with First-Line Chemotherapy”

**DOI:** 10.3390/ijms18061308

**Published:** 2017-06-19

**Authors:** Chiara Nicolazzo, Cristina Raimondi, Flavia Loreni, Paola Gazzaniga, Angela Gradilone

**Affiliations:** Circulating Tumor Cells Unit, Department of Molecular Medicine, Sapienza University of Rome, Viale Regina Elena, 324, 00161 Rome, Italy; chiara.nicolazzo@uniroma1.it (C.N.); cristina.raimondi@libero.it (C.R.); flavialoreni91@gmail.com (F.L.); paola.gazzaniga@uniroma1.it (P.G.); angela.gradilone@uniroma1.it (A.G.)

## To the Editor,

We read with great interest the article “Circulating Cell-Free DNA and Circulating Tumor Cells as Prognostic and Predictive Biomarkers in Advanced Non-Small Cell Lung Cancer Patients Treated with First-Line Chemotherapy” published by Coco, S. et al. in *International Journal of Molecular Sciences* on 11 May 2017 [1]. The aim of the authors was to evaluate the prognostic role of cell-free tumor DNA (cfDNA) quantification and circulating tumor cells (CTCs) enumeration, separately or conjunctionally, in non-small cell lung cancer (NSCLC) patients receiving first line chemotherapy. Based on their data, the authors concluded that cfDNA demonstrated a more reliable biomarker than CTCs in the overall population. However, the CTC analysis was performed based on morphological characteristics, such as nuclear size, nuclear/cytoplasmic ratio, hyperchromatic nucleus and nuclear membrane irregularities. In this regard, we would like to make a few comments, which are mainly technical in nature. 

The authors chose a device (ScreenCell Cyto, ScreenCell, Sarcelles, France) which is simple, inexpensive and allows to study the biological characteristics of CTCs isolated from 3 ml of blood. Nevertheless, Haematoxylin–Eosin (H&E) staining alone does not allow a correct enumeration of CTCs, which would necessitate further molecular characterization for cancer-specific biomarkers, such as, in this case, thyroid transcription factor 1 (TTF-1) for adenocarcinomas and high-molecular weight (HMW) keratins (CK5/6) for squamous-cell carcinomas [2]. 

Indeed, as early as 2013, El-Heliebi et al. reported that reliable detection of CTCs should be confirmed by immunocytochemical and/or molecular biological methods [3]. Thus, beyond the tumor cells’ heterogeneity, it is conceivable that a leukocyte subpopulation is present among the cells isolated on the filter and could lead to a CTC number overestimation in the present work. 

Furthermore, the association between a higher number of CTCs and better prognosis is definitely uncommon and would deserve further discussion. Our feeling is that, similarly to what is reported by Juan et al. [4], the authors should re-consider that the use of H&E staining alone, as the only criterion for CTCs identification, may lead to an overestimation of CTC number in their clinical records.

As a suggestion, the authors could perform a leukocytes depletion before detecting CTCs by using a ScreenCell Cyto kit [5] and subsequently characterize such cells through a multiplex immunofluorescence for 4′,6-diamidino-2-phenylindole (DAPI), TTF-1 and HMW keratins, to discriminate the real CTCs from other cells, followed by H&E staining. This is the only way to confirm the specificity and the sensibility of the morphological examination after H&E assay. 

We hope that our observation and advice will be accepted as a productive analysis.
